# QSAR, Docking, and Molecular Dynamics Simulation Studies of Sigmacidins as Antimicrobials against *Streptococci*

**DOI:** 10.3390/ijms23084085

**Published:** 2022-04-07

**Authors:** Jiqing Ye, Xiao Yang, Cong Ma

**Affiliations:** 1 State Key Laboratory of Chemical Biology and Drug Discovery, and Department of Applied Biology and Chemical Technology, The Hong Kong Polytechnic University, Kowloon, Hong Kong, China; ji-qing.ye@polyu.edu.hk; 2 Department of Microbiology, Prince of Wales Hospital, The Chinese University of Hong Kong, Shatin, Hong Kong, China

**Keywords:** *Streptococci*, antimicrobials, QSAR, MD simulation

## Abstract

*Streptococci* are a family of bacterial species significantly affecting human health. In addition, environmental *Streptococci* represent one of the major causes of diverse livestock diseases. Due to antimicrobial resistance, there is an urgent need for novel antimicrobial agent discovery against *Streptococci*. We discovered a class of benzoic acid derivatives named sigmacidins inhibiting the bacterial RNA polymerase-σ factor interaction and demonstrating excellent antimicrobial activity against *Streptococci*. In this work, a combinational computer approach was applied to gain insight into the structural basis and mechanism of action of sigmacidins as antimicrobials against *Streptococcus pneumoniae*. Both two- and three-dimensional quantitative structure-active relationships (2D and 3D QSAR) of sigmacidins displayed good predictive ability. Moreover, molecular docking and molecular dynamics simulation studies disclosed possible contacts between the inhibitors and the protein. The results obtained in this study provided understanding and new directions to the further optimizations of sigmacidins as novel antimicrobials.

## 1. Introduction

*Streptococci* are a large family of *Streptococcus* species widely present in the environment and as microbiota of mammals such as humans, wild animals and livestock. *Streptococcus pneumoniae*, belonging to the alpha-hemolytic streptococcal species, is one of the most common human pathogens leading to a range of pneumococcal diseases, including otitis media, sinusitis, pneumonia, septicemia, and meningitis [1]. The beta-hemolytic streptococcal species such as *Streptococcus pyogenes* (Group A *Streptococcus*, GAS) and *Streptococcus agalactiae* (Group B *Streptococcus*, GBS) represent the other two pathogens frequently triggering human diseases such as streptococcal pharyngitis (strep throat), impetigo, pneumonia, and meningitis [2,3]. In addition, more than one third of herd mastitis incidences are caused by “Environmental *Streptococci*” [4,5]. This name was coined after *Streptococci* which leads to animal diseases [6], such as *Streptococcus dysgalactiae*, *Streptococcus uberis* (Groups C and G *Streptococci*, GCS and GGS), and *Enterococcus* spp., which used to be classed in the genus *Streptococcus* (Group D *Streptococcus*) prior to 1984 [7]. The medical application to treat streptococcal infections by antibiotics is sometimes ineffective due to antimicrobial resistance [5,8]. As a result, *S. pneumoniae* has been listed in the World Health Organization Global Priority Pathogens List for which new antibiotics are urgently needed [9].

**Figure 1:**
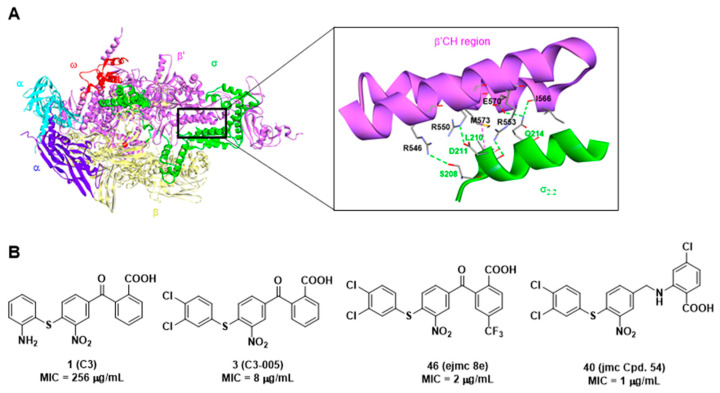
(**A**) *Escherichia coli* RNA polymerase holoenzyme (α_2_ββ′ωσ) (PDB: 1IW7, [17]) and the detailed interactions between β′CH and σ_2.2_, hydrogen bonds: green, hydrophobic: magenta; (**B**) Chemical structures of reported sigmacidins derivatives targeting the RNAP-σ interaction and their activities against *S. pneumoniae*.

The emergence of multidrug resistance to current antibiotics among pathogens highlights the importance of the discovery of novel antimicrobials with minimized antimicrobial resistance. Protein-protein interactions (PPI) are appropriate targets for reducing antimicrobial resistance [10]. We focused on the specific and conserved bacterial PPIs for antimicrobial discovery [11]. Bacterial RNA polymerase (RNAP) comprises several subunits: 2α, β, β′, ω (Gram-positive bacteria include one more subunit ε) [12,13], and interacts with the bacteria-specific transcription factor σ to form a holoenzyme to recognize DNA promoters and initiate bacterial transcription. Therefore, this essential PPI represents an appropriate target for antimicrobial agent discovery [14]. Biochemistry studies have shown that the binding site between the region 2.2 of σ factor (σ_2.2_) and the clamp helix region of the RNAP β′ subunit (β′CH) is the major binding site for this PPI [15]. Based on the structural biological information [16,17] (Figure 1A), we designed a pharmacophore model and screened out three hit compounds (**C3**, **C4**, and **C5**) showing the specific inhibitory activity [18]. The optimization of compound **1** (**C3**) resulted in the discovery of a set of derivatives such as **3** (**C3-005**), **46** (ejmc **8e**), and **40** (jmc Cpd. **54**) with dramatically improved antibacterial activities, in particular against *Streptococci* including *S. pneumoniae*, *S. pyogenes*, *S. agalactiae*, and *Enterococcus faecalis* with minimum inhibitory concentrations (MIC) lowered to 1 μg/mL, comparable to current antibiotics in the market (Figure 1B) [19,20,21]. Considering the general structure of benzoic acid in this class of compounds and the protein σ factor on which these compounds mimic for binding, we named this class of antimicrobials as “sigmacidin”.

With a new class of antimicrobial agents in hand, we intend to make use of the quantitative structure-activity relationship (QSAR) analysis to explore the relationship between the observed antimicrobial activity and numerical descriptors in order to predict the biological properties of perspective compounds and guide future syntheses [22]. The 2D QSAR study considers physicochemical properties of signal atoms and functional groups and their contribution to biological activity, while 3D QSAR could foresee the potential three-dimensional structure of the ligand molecules [23]. In this study, statistical methods including multiple linear regression (MLR) and partial least square analysis (PLS) were applied to analyze the correlation between properties or descriptors of the molecules and molecular properties [24].

In an attempt to reveal the relationships between the chemical structures and their activity against the representative *S. pneumoniae*, we took fiftysix molecules reported previously [20,21] to generate a set of quantitative rules and construct both 2D and 3D QSAR models for the design of novel derivatives. Their structures are given in the supporting information (Table S1) and their activities against *S. pneumoniae* (MIC) are shown in Table 1. Moreover, molecular docking and molecular dynamics (MD) simulations were performed to gain insight into the structural basis and the inhibitory mechanism of the inhibitors.

## 2. Results and Discussion

### 2.1. Two-Dimensional QSAR

#### 2.1.1. Two-Dimensional QSAR Study

A 2D QSAR study for compounds against *S. pneumoniae* was performed to determine the factors/descriptors related to the antibacterial activities of compounds **1**–**56**, and to disclose the structural features contributing towards the bacterial inhibitory activities. In this study, the molecular properties for the compounds in the training set were calculated using the “Calculate Molecular Properties” protocol in Discovery Studio 2016 (DS 2016).

Descriptors used for the building of the model were selected based on the results of the intercorrelation matrix between the calculated descriptors. In the present research, the selected descriptors have intercorrelation values lower than 0.5 (Table 2) to avoid model overfitting, and the result of the matrix analysis revealed the independence of these descriptors.

A Multiple Linear Regression (MLR) analysis method was used to construct the model. The statistical quality of the MLR model was judged by the calculation of the squared correlation coefficient (r^2^) for internal validation and the predictive squared correlation coefficient (r^2^_pred_) for external validation [25]. Moreover, the predictive power of the QSAR model was verified using LOO internal validation or cross validation (q^2^). Usually, a value of q^2^ > 0.5 is considered acceptable [26]. In this model, the r^2^ was 0.732, r^2^_pred_ was 0.613, and q^2^ equaled 0.562, which indicated the true predictive ability of the model (Table 3)

#### 2.1.2. Two-Dimensional QSAR Model Analysis

**Figure 2:**
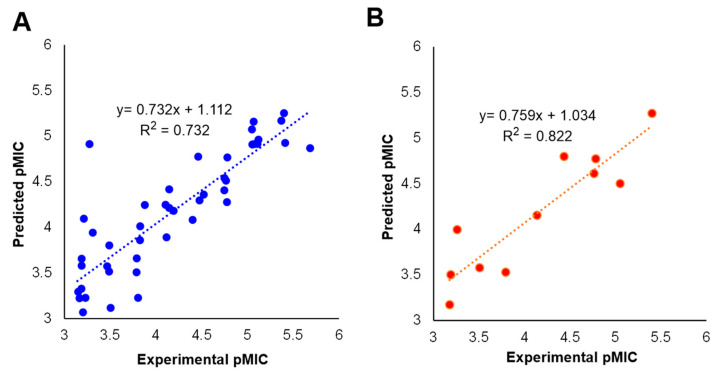
Predicted versus experimental pMIC of (**A**) the training set and (**B**) the test set.

The predicted activities for the inhibitors versus their experimental activities and the residues between them are listed in Table 1. The correlation between the predicted activities and the experimental activities are depicted in Figure 2. These results demonstrated that the predicted activities by the constructed MLR model were in good agreement with the experiment data, suggesting that the 2D QSAR model was reliable for structure-activity prediction.

Equation (1) represents the MLR model obtained by DS 2016. According to Equation (1), four descriptors, including (a) ALogP reflecting lipophilicity [27], (b) hydrogen bond acceptor (HBA) which is critical to potency, selectivity, permeability, and solubility [28], (c) molecular polar surface area, a guideline towards the improvement of oral absorption and permeability [29], and (d) LUMO eigenvalue that is related to electrostatic properties [30], were used to describe the relationship between chemical properties and the antimicrobial activity. Compared with the molecular polar surface area, AlogP, HBA count, and LUMO Eigenvalue showed higher correlations, and slight variations of these descriptors significantly affected the activity.

Equation (1) representing the 2D QSAR model:

pMIC = 0.5647 + 0.5705(ALogP) − 0.2073(HBA Count) + 0.0090 (Molecular Polar Surface Area) + 0.2049(LUMO Eigenvalue VAMP)(1)

To further improve the predictive ability of the above model, an outlier analysis was conducted using the Find Outlier Molecules module of DS 2016 to identify the outliers in the dataset, and the acceptable level was set to 95 (95% confidence interval). Results showed that four compounds were returned as outliers, including compounds **10**,for which the Molecular PSA was too high, **33** for which the LUMO Eigenvalue VAMP was too low, and **39** and **43**, for which the Molecular PSA was too low. These four compounds were removed and a new 2D QSAR model was constructed. In the revised model, r^2^ was 0.777, r^2^_pred_ was 0.721, and q^2^ equaled to 0.690, which indicated the improved predictive ability of the model (Table 4). 

Equation (2) represents the revised MLR model. As shown in the equation, changes in the ALogP and LUMO Eigenvalue VAMP may affect the antimicrobial active significantly as they have higher correlations in comparison with HBA count and molecular polar surface area. 

Equation (2) representing the upgraded 2D QSAR model:

pMIC = 0.6578 + 0.5757(ALogP) − 0.07427(HBA Count) + 0.01056 (Molecular Polar Surface Area) − 0.2082(LUMO Eigenvalue VAMP)(2)

### 2.2. Three-Dimensional QSAR

#### 2.2.1. Molecular Alignment

**Figure 3:**
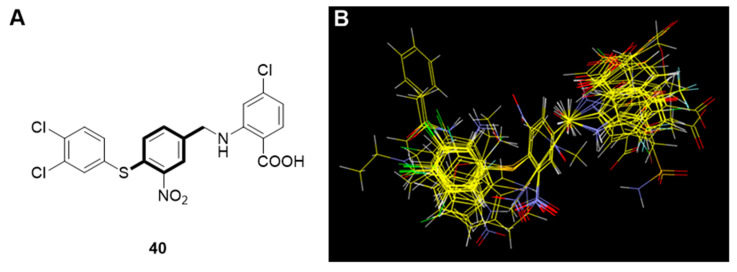
(**A**) Template used for molecular alignment of the PPI inhibitors (Compound **40** with the common motif bolded); (**B**) alignment of molecules on compound **40**.

Structural alignment of the molecules is critical to both the predictive accuracy of a 3D QSAR model and reliability of contour models. Therefore, we applied flexible alignment to align all the molecules in this study. The most active compound, **40**, was selected as the alignment template and the rest of the compounds were aligned to it by using the common substructure as displayed in Figure 3.

#### 2.2.2. Three-Dimensional QSAR Study

The 3D QSAR model in this study was built by the Field-Based model module in Maestro 10.2. The statistical parameters are presented in Table 5. Here, r^2^ is the non-cross-validated value for the regression, r^2^_CV_ is the LOO cross-validated correlation value, r^2^ scramble represents the average value of r^2^ from a series of models built using scrambled activities, and “Stability” reflects the sensitivity of the model to omissions from the training set. Q^2^ is directly analogous to r^2^, but is based on the test set predictions. When the r^2^ value is larger than the stability value, this is an indication that the dataset is over-fit. As we set PLS factor to six considering the statistical results, the three-factor model was selected with r^2^ and r^2^ _CV_ values of 0.805 and 0.568, respectively, and a stability value of 0.883 (Table 5).

The model was built using four fields, including steric, electrostatic, hydrogen bond (H-bond) donor, and H-bond acceptor. As shown in Table 6, the steric field and the hydrophobic field contributed significantly to the antibacterial activity with percentages of 36.1% and 29.8%, respectively.

**Figure 4:**
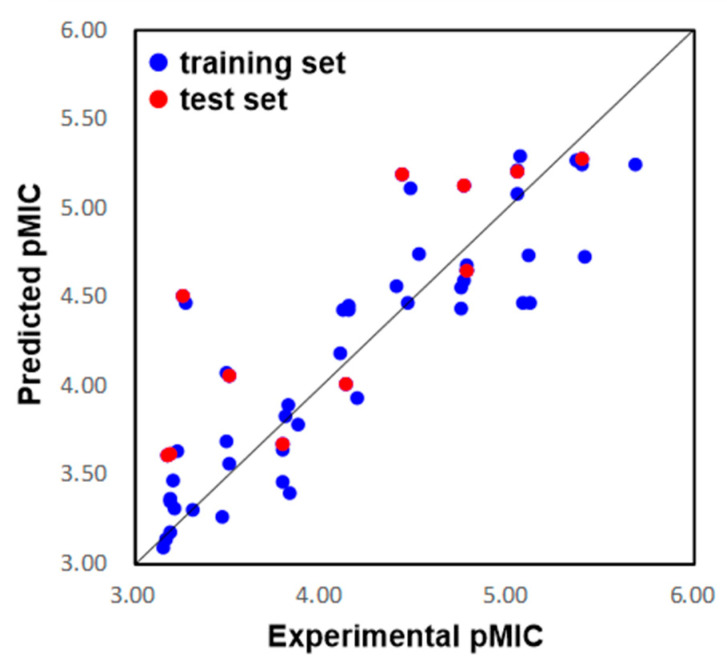
Plots of the predicted pMIC values by the 3D-QSAR model versus the observed pMIC values.

The eleven compounds randomly selected by Maestro 10.2 were used as the test set to validate the predictive ability of the 3D QSAR model. As a result, the predicted pMIC values shown in Table 1 were in good alignment with the experimental data, with a Pearson-r (the correlation between the predicted and observed activity for the test set) value of 0.835 and a Q^2^ value of 0.528. The correlation plots between the experimental and predicted pMIC values for both the training and test sets were shown in Figure 4. Though some outliers may possibly be generated, the results demonstrated the potential of the 3D QSAR model to be used for drug design with a good predictive power.

#### 2.2.3. Interpretation of the 3D QSAR Contour Maps

**Figure 5:**
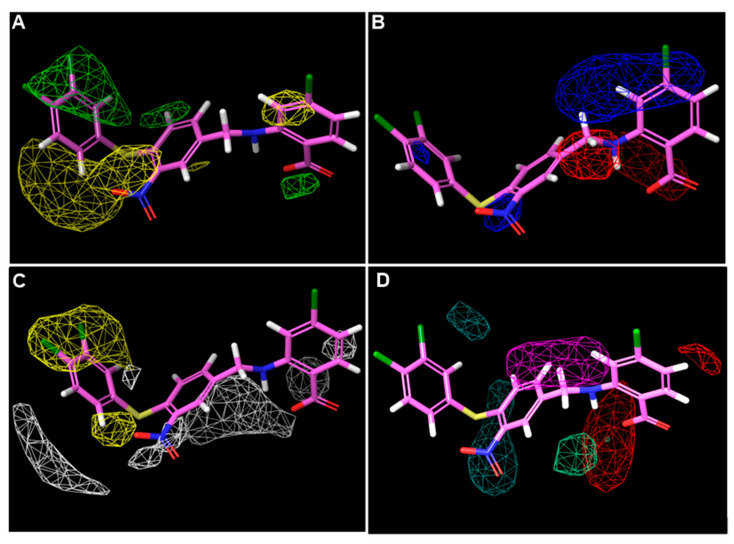
Three-dimensional QSAR StDev*Coeff contour maps using the most bioactive compound **40**. (**A**) Steric fields: favored (green) and disfavored (yellow); (**B**) electrostatic fields: electropositive (blue) and electronegative (red); (**C**) hydrophobic field: favored (yellow) and disfavored (white); (**D**) H-bond acceptor field: favored (Red) and disfavored (magenta); Hydrogen bond donor field: favored (blackish green) and disfavored (pale green).

To visualize the structure-activity relationship of these inhibitors, the steric, electrostatic, hydrophobic, H-bond donor, and H-bond acceptor contour maps of the models are displayed in Figure 5. The most active compound, **40**, was used for further analysis.

In the steric contour (Figure 5A), the green regions represented that the introduction of bulky substituents might increase activity, while steric hindrance should be avoided in the yellow regions. As shown in Figure 5A, a relatively large green contour was found around 3,4-diCl groups of compound **40**, indicating that bulky substituents might be preferred in this region, while at 5- and 6-positions of the right benzene ring, steric hindrance was unfavorable.

The electrostatic contour (Figure 5B) for compound **40** showed that a relatively large blue contour was located around the *para*-position to the -COOH group, suggesting that electron-deficient substituents may increase the activity. In addition, a small blue region was found to surround the -NO_2_ group, this can explain why reduction or removal of -NO_2_ resulted in reduction of the antimicrobial activity. In contrast, the red contour was mainly located around the linker, which meant electron-rich linkers may improve the activity.

On the hydrophobic contour map (Figure 5C), the yellow regions indicated that the hydrophobic groups were preferred, while the white regions favored hydrophilic groups. It was shown that a relatively large blue region appeared around the 3,4-diCl groups; together with the prediction of the steric contour, the models indicated that replacing the -Cl with bulky and hydrophobic substituents might be beneficial to the antibacterial activity. In the contrast, the white regions were adjacent to the molecule but did not wrap it up like the yellow regions.

H-bond acceptor and donor contour maps are displayed in Figure 5D. H-bond acceptors were favored as red regions and unfavored in the magenta regions. Moreover, H-bond donors were preferred as blackish green and pale green as the unfavorable regions. The magenta and blackish green contours covered the middle benzene ring, while the red and pale green regions encircled the -COOH group. Briefly, the H-bond acceptor and donor groups contributed less to the activity compared to the steric and hydrophobic groups. 

### 2.3. Docking and MD Simulations Studies of Compound ***1*** and ***40***

#### 2.3.1. Docking Studies

**Figure 6:**
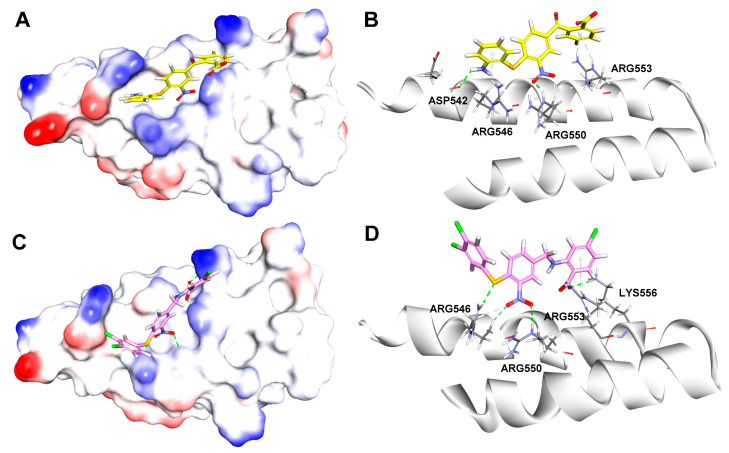
Binding modes and details of interaction of compound **1** (yellow, (**A**,**B**)) and compound **40** (magenta, (**C**,**D**)) in complex with β′CH extracted from RNAP holoenzyme (PDB: 1IW7, [17]).

To compare the differences between the most bioactive compound **40** and the hit compound **1** in the binding processes, their potential binding modes and key interactions were analyzed using the LibDock module of DS 2016. As shown in Figure 6A,B, the -NH_2_ and -NO_2_ groups of compound **1** formed hydrogen bonds with Asp542 (H···O 2.15 Å) and Arg550 (O···H 1.85 Å) of β′CH region, respectively. Additionally, some weak π-cation interactions existed between the aromatic ring of compound **1** and Arg553 and Arg546.

In Figure 6C,D, it is suggested that compound **40** formed three classical hydrogen bonds with β′-CH region, including two H-bonds between the carboxylic group and Arg553 (O···H 2.68 Å) and Lys556 (O···H 2.32 Å), one H-bond between -NO_2_ and Arg550 (O···H 2.93 Å), while the S atom formed a nonclassical H-bond with Arg546 (S···H 2.48 Å). 

The docking results indicated both compounds made extensive contacts with β′CH. In comparison to the hit compound **1**, compound **40** made more interactions with the “hotspot” residues, including Arg546, Arg550, and Arg553 through hydrogen bonds.

#### 2.3.2. MD Simulation Studies

**Figure 7:**
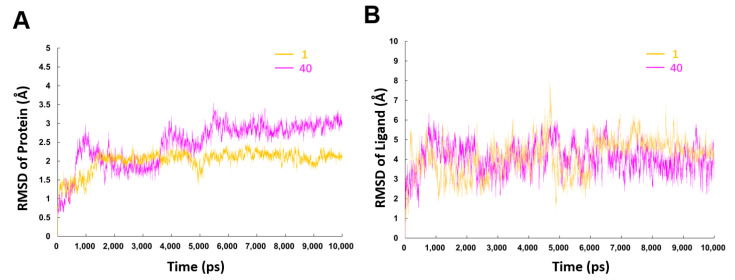
RMSD values of backbone atoms of the protein (**A**) and the heavy atoms in the ligands (**B**) for the compounds **1**- and **40**-β′CH systems as a function of the simulation time.

To further understand the difference on the binding processes of hit compound **1** and compound **40**, 10 ns MD simulations based on the above-mentioned binding modes were performed. To study the dynamic stability of both systems, root-mean-square deviations (RMSD) from the starting structures were analyzed (Figure 7). The plots showed that both the two systems reached equilibrium within 6 ns, and the proteins and ligands in both systems were stable after equilibrium. Average RMSD values for the protein and ligand in **1-**β′CH bound system were 2.0 Å and 4.1 Å, respectively, while the corresponding values for the **40**-β′CH bound system were 2.3 Å and 4.0 Å, respectively. Moreover, it was observed that compound **40** fluctuated more violently which might be due to the distances of the connected bonds between **40** and β′CH in the docking model. They were slightly longer than the distances between compound **1** and β′CH. In addition, the protein in the 40-β′CH system encountered more sizable rearrangement. This may be due to compound **40** having a more flexible structure. It could generate more conformations which require the protein to make more changes to adapt.

**Figure 8:**
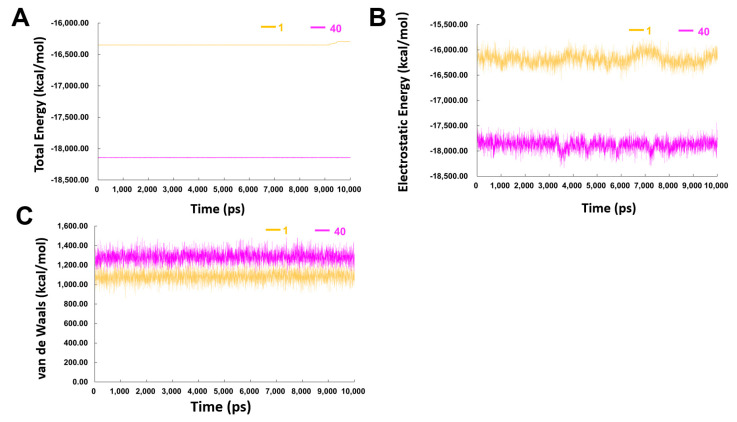
(**A**) Total energy (**B**) electrostatic energy and (**C**) van de Waals energy of the complexes of compounds **1**− and **40**−β′ CH region in the 10 ns MD simulations.

The energy of both complexes through MD simulation is shown in Figure 8. During the 10 ns production run, due to the existence of counter ions, the potential energy will often not decrease [31]. Results demonstrated that compound **40** in complex with the β′CH region had a much lower total energy compared to that of hit compound **1** (Figure 8A), especially the electrostatic energy (Figure 8B), while the *van der Waals* energies of the two systems were similar (Figure 8C). These results indicated that more attention should be given on the electrostatic energy when developing high-affinity inhibitors of the β′CH-σ interaction.

The binding energy of both inhibitors with β′CH were calculated using the Calculate Binding Energy module of DS 2016. For each system, binding energy calculation was performed for snapshots extracted every 100 ps from the last 2 ns of the whole 10 ns MD trajectory. For each snapshot, the free energy was calculated for each molecular species (complex, protein, and ligand), and the binding free energy was defined as: ΔE_binding_ = ΔE_Complex_ − ΔE_Receptor_ − ΔE_Ligand_ [32]. Results showed that the average binding energy for compounds **1** and **40** were −12.1359 kcal/mol and -9.8806 kcal/mol, respectively. These results showed similar binding interactions of compounds, while compound **40** is a more flexible molecule, which may lead to higher binding energy as demonstrated.

**Figure 9:**
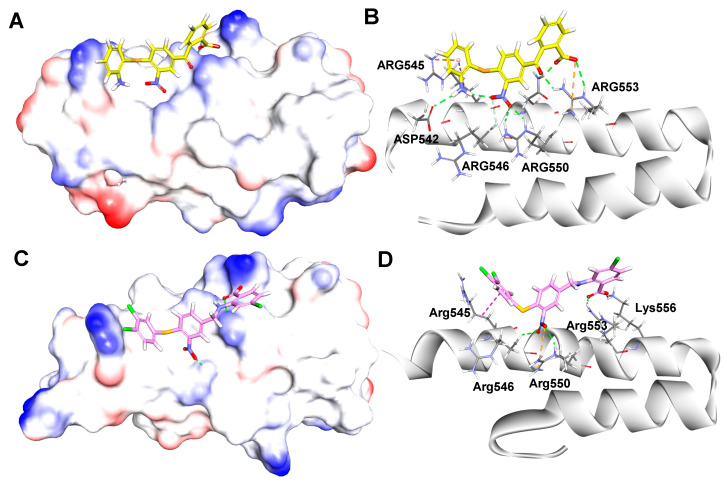
(**A**) Snapshot at 10 ns showed the binding mode of compound **1** in complex with β′CH region, and details of the interactions (**B**); (**C**) snapshot at 10 ns showed the binding mode of compound **40** in complex with β′CH region, and details of interactions (**D**). Hydrogen bonds (green), electrostatic interactions (yellow) and hydrophobic interactions (magenta).

To further understand the mechanic of action of the inhibitors, the final snapshots of the 10 ns trajectory were used to analyze the interactions between β′CH and compound **40** and **1**, respectively. For compound **1**, in comparison with the starting conformation, the most outstanding difference was that the benzoic acid moiety was turned over to form two hydrogen bonds between the carboxylic group and Arg549 and Arg553 (Figure 9A,B). Moreover, the nitro group not only retained the H-Bonding interaction with Arg550, but also formed a new hydrogen bond with Arg546. 

For compound **40**, the three benzene rings of compound **40** positioned much closer to the surface of the helix. This led to the molecule that made more interactions with the key residues of β′CH region (Figure 9C). As shown in Figure 9D, the carboxylic acid group of **40** formed a hydrogen bond and a salt-bridge with residues Arg553 and Lys556, respectively. Moreover, the nitro group formed two hydrogen bonds with Arg546 and Arg550, while a salt bridge was also formed between the nitro group and Arg550. In addition, the 3,4-diCl group of the left benzene ring formed hydrophobic interactions with Arg545. Overall, these interactions may play key roles for the bioactivity of compound **40**. 

## 3. Materials and Methods

### 3.1. Dataset

All small molecule RNAP-σ inhibitors and their antimicrobial activities (MIC, μg/mL) were adopted from previous studies [20,21]. The MIC values in units of microgram per milliliter (μg/mL) were transformed in molarity (M) and subsequently transformed to pMIC (−logMIC). The dataset was divided into a training set for model generation and a test set (Table 1) for model validation, containing 45 and 11 compounds, respectively. The test set was chosen randomly by Maestro 10.2.

### 3.2. Preparation of the Small Molecules

The 3D structures of compounds were generated using Maestro 10.2, geometrically minimized with Macromodel (Maestro 10.2) based on the OPLS-2005 force field and all other parameters were set to the default settings [33].

### 3.3. Two-Dimensional QSAR Model Construction

Two-dimensional molecular properties of the training set compounds were calculated by module “Calculate Molecular Properties” in DS 2016. Two-dimensional descriptors including PKa, AlogP, molecular weight, molecular property counts (Num_aromatic Rings, Num_H_Acceptors, Num_H_Donors, Num_Rings, Num_RotatableBonds), Molecualr surface Area, Molecular_Fractional Polar Surface Area, HOMO Eigenvalue VAMP, and LUMO Eigenvalue VAMP were adopted. The model was validated using the test set correlation and Leave-one-out (LOO) cross validation. 

### 3.4. Three-Dimensional QSAR Model Construction

A 3D QSAR model was developed by Maestro 10.2. The alignment was achieved by using the Flexible Ligand Alignment module. The 3D QSAR model was generated by Field-based QSAR module with default parameters. The field style was set to Gaussian field, including steric, electrostatic, hydrophobic, H-bond acceptor, and donor field. The maximum PLS factor was set to six and in the PLS regression analysis, a leave-one-out (LOO) cross validation was performed to find the optimal number of components. The descriptors were generated in a 3D cubic lattice with grid spacing of 1 Å and extending to 3 Å units beyond the aligned molecules in all directions. In addition, the cutoff values for truncating steric force and electrostatic force fields were both set to 30 kcal/mol. 

### 3.5. Docking and Molecular Dynamic Simulations

The crystal structure of the β′CH region was extracted from the crystal structure of bacterial RNAP (PDB: 1IW7, [17]) which was downloaded from Protein Data Bank. Structures of the compounds and the protein for docking were imported to DS 2016 and the conformations were generated with the protocol “Prepare Protein” and “Prepare Ligands”, respectively. Molecular Docking was performed using the LibDock tool and the identified critical residues for the σ_2.2_-β′CH region PPI (including Arg546, Arg550, Arg553, Leu566) were defined as the binding sites. The docking process was conducted with the default parameters unless otherwise mentioned. MD simulation was conducted in a similar manner as described [34]. Binding free energy was calculated according to the literature [32].

## 4. Conclusions

*Streptococci* are an important bacterial family closely related to human wellbeing, while environmental *Streptococci* significantly affect herd health. Antimicrobial resistance to conventional antibiotics is emerging due to natural resistance mechanisms and antibiotic misuse. Therefore, novel antimicrobial agents are urgently required. We focused on bacterial transcription [35,36] and discovered a series of benzoic acid derivatives and named them sigmacidins. They were capable of mimicking bacterial transcription factor σ at the region 2.2 to disrupt its binding to RNAP and to exhibit excellent antimicrobial activity against *Streptococci* including *E. faecalis*.

In this study, a combined computational approach was applied to investigate the relationship of the structural basis and antimicrobial activities of sigmacidins. Both 2D and 3D QSAR models were constructed, and the binding poses of the inhibitors to the protein were obtained.

The 2D QSAR model constructed revealed close structure-activity correlation and contribution of various properties/descriptors in the activity. In the model identified in this study, ALogP, hydrogen bond acceptor (HBA), molecular polar surface area, and LUMO eigenvalue were taken to describe the SAR. The 2D QSAR equation implied that the activity of the compounds was related to and can be improved by increasing AlogP, molecular polar surface area, and the LUMO eigenvalue. This equation will be useful to estimate antimicrobial activity of newly designed compounds. Two-dimensional QSAR for bioactivity prediction is simple and efficient; however, it was obtained based on limited structures of substituents and may have accurate correlations to a relatively small range of substitutions for further structural optimizations. 

The 3D QSAR model gained further insight into the 3D structure information for the understanding of the SAR of these antimicrobials. The importance of the steric and hydrophobic properties of the 3,4-substitution of the left benzene ring was highlighted, while the substituents at *para*-position of the -COOH group could be further explored for novel derivative synthesis. Besides guiding the modifications of existing molecules, the constructed model can also be used directly for virtual screening to identified novel hits.

Finally, molecular docking indicated possible binding poses of the inhibitors in complex with β′CH and MD simulations used to rationalize the docked poses. In this study, the docking model of the **40**-β′CH system showed similar stability to **1**-β′CH and the binding free energy of **40**-β′CH was slightly higher than that of **1**-β′CH, probably due to its flexible structure. Fortunately, the surface of the protein fragment is enriched in arginine which is elastic and able to accommodate more conformational changes of compound **40**. In addition, compared to the starting docking models, after 10 ns simulation, both compounds formed more H-bonding contacts with β′CH region, especially with “hotspot” residues, including Arg546, Arg550, and Arg553. This indicated that the binding affinity might be increased by enhancing acidity of the inhibitors. For example, the nitro group can be replaced by acidic substituents. While compound **40** demonstrated significantly superior antibacterial activity to compound **1**, the possible reasons may include the greater bacterial cell membrane permeability of compound **40** which was optimized from hit compound **1**. Nevertheless, the docking and MD simulations showed some difference, probably due to the challenging PPI target with a relatively flat binding site. Here, we need to combine the two methods which put forward possible contacts between ligands and the β′CH region. This combination is useful for future structure-based drug design. The newly designed compounds that fulfill the requirement by these 3D features may be able to bind to the same target protein and possess significant antimicrobial activity, which remain to be experimentally evaluated.

Overall, the models established in this study provided useful indications for the design of novel sigmacidins derivatives against pathogenic *Streptococci*. Note that sigmacidins also demonstrated excellent antimicrobial activity against *Staphylococci* such as *Staphylococcus aureus*, *Staphylococcus epidermidis*, and *Staphylococcus Saprophyticus* [20,21]. We believe that the further development of sigmacidins via ligand-based and structure-based drug design will contribute to novel antimicrobial agent discovery in the post-antibiotic era.

## Figures and Tables

**Table 1:** Compounds selected for modeling and their observed and predicted activity against *S. pneumoniae*.

Cpd.	MIC (μg/mL)	MW	MIC (M)	pMIC	2D QSAR		3D QSAR	
					Predicted pMIC	Δ ^b^	Predicted pMIC	Δ ^b^
**1 ^a^**	256	394.40	6.49 × 10^−4^	3.188	3.503	−0.315	3.616	−0.428
**2 ^a^**	128	409.41	3.13 × 10^−4^	3.505	3.579	−0.074	4.060	−0.555
**3**	8	448.28	1.78 × 10^−5^	4.748	4.406	0.342	4.552	0.196
**4**	256	362.34	7.07 × 10^−4^	3.151	3.295	−0.144	3.089	0.062
**5**	256	354.36	7.22 × 10^−4^	3.141	2.899	0.242	2.994	0.148
**6**	128	409.42	3.13 × 10^−4^	3.505	3.119	0.386	3.564	−0.059
**7 ^a^**	64	395.43	1.62 × 10^−4^	3.791	3.529	0.262	3.674	0.117
**8**	64	394.40	1.62 × 10^−4^	3.790	3.508	0.282	3.642	0.148
**9**	128	394.40	3.25 × 10^−4^	3.489	3.516	−0.027	3.692	−0.203
**10**	256	523.52	4.89 × 10^−4^	3.311	3.942	−0.631	3.303	0.008
**11**	256	376.36	6.80 × 10^−4^	3.167	3.224	−0.057	3.140	0.027
**12**	256	394.35	6.49 × 10^−4^	3.188	3.328	−0.14	3.181	0.007
**13**	128	376.36	3.40 × 10^−4^	3.468	3.575	−0.107	3.261	0.207
**14**	8	449.31	1.78 × 10^−5^	4.749	4.541	0.208	4.437	0.313
**15 ^a^**	256	463.29	5.53 × 10^−4^	3.258	3.996	−0.738	4.507	−1.249
**16**	256	418.44	6.12 × 10^−4^	3.213	4.095	−0.882	3.311	−0.098
**17**	128	397.38	3.22 × 10^−4^	3.492	3.802	−0.31	4.072	−0.580
**18 ^a^**	32	436.48	7.33 × 10^−5^	4.135	4.154	−0.019	4.012	0.123
**19**	256	409.42	6.25 × 10^−4^	3.204	3.069	0.135	3.467	−0.263
**20**	64	409.42	1.56 × 10^−4^	3.806	3.229	0.577	3.827	−0.021
**21**	32	407.44	7.85 × 10^−5^	4.105	4.247	−0.142	4.181	−0.076
**22 ^a^**	256	383.38	6.68 × 10^−4^	3.175	3.173	0.002	3.612	−0.437
**23**	32	448.28	7.14 × 10^−5^	4.146	4.418	−0.272	4.426	−0.280
**24**	32	498.51	6.42 × 10^−5^	4.193	4.182	0.011	3.936	0.256
**25**	64	428.85	1.49 × 10^−4^	3.826	3.860	−0.034	3.894	−0.068
**26**	64	431.23	1.48 × 10^−4^	3.829	4.013	−0.184	3.401	0.427
**27**	64	395.43	1.62 × 10^−4^	3.791	3.660	0.131	3.462	0.329
**28**	256	395.43	6.47 × 10^−4^	3.189	3.581	−0.392	3.366	−0.177
**29**	256	436.44	5.87 × 10^−4^	3.232	3.228	0.004	3.632	−0.400
**30**	4	483.75	8.27 × 10^−6^	5.083	4.915	0.168	4.469	0.614
**31**	16	479.33	3.34 × 10^−5^	4.477	4.297	0.180	5.112	−0.635
**32**	16	534.41	2.99 × 10^−5^	4.524	4.359	0.165	4.744	−0.221
**33**	64	484.38	1.32 × 10^−4^	3.879	4.246	−0.367	3.787	0.092
**34**	16	463.33	3.45 × 10^−5^	4.462	4.776	−0.314	4.466	−0.004
**35**	256	396.78	6.45 × 10^−4^	3.190	3.656	−0.466	3.353	−0.163
**36**	4	528.20	7.57 × 10^−6^	5.121	4.962	0.159	4.467	0.654
**37**	256	483.75	5.29 × 10^−4^	3.276	4.912	−1.636	4.470	−1.194
**38 ^a^**	4	449.31	8.90 × 10^−6^	5.050	4.500	0.550	5.204	−0.154
**39**	16	403.28	3.97 × 10^−5^	4.401	4.082	0.319	4.561	−0.160
**40**	1	483.75	2.07 × 10^−6^	5.685	4.868	0.817	5.246	0.439
**41 ^a^**	16	434.29	3.68 × 10^−5^	4.434	4.798	−0.364	5.189	−0.755
**42**	32	418.29	7.65 × 10^−5^	4.116	3.891	0.225	4.428	−0.311
**43**	32	446.35	7.17 × 10^−5^	4.145	4.213	−0.068	4.449	−0.304
**44 ^a^**	8	482.72	1.66 × 10^−5^	4.781	4.773	0.008	4.648	0.133
**45**	8	482.72	1.66 × 10^−5^	4.781	4.766	0.015	4.653	0.128
**46**	2	516.27	3.87 × 10^−6^	5.412	4.925	0.487	4.725	0.687
**47**	4	468.74	8.53 × 10^−6^	5.069	5.159	−0.090	5.289	−0.221
**48**	4	448.32	8.92 × 10^−6^	5.050	5.073	−0.023	5.081	−0.031
**49**	8	478.30	1.67 × 10^−5^	4.777	4.278	0.499	4.681	0.096
**50**	8	466.27	1.72 × 10^−5^	4.766	4.512	0.254	4.592	0.174
**51**	4	516.27	7.75 × 10^−6^	5.111	4.914	0.197	4.737	0.374
**52 ^a^**	2	502.29	3.98 × 10^−6^	5.400	5.271	0.129	5.273	0.126
**53 ^a^**	8	464.32	1.72 × 10^−5^	4.764	4.611	0.153	5.126	−0.362
**54**	2	468.74	4.27 × 10^−6^	5.370	5.169	0.201	5.271	0.099
**55**	4	452.28	8.84 × 10^−6^	5.053	4.908	0.145	5.218	−0.164
**56**	2	502.29	3.98 × 10^−6^	5.400	5.251	0.149	5.243	0.157

^a^ Compounds taken for the test set; ^b^ **Δ** = Experimental pMIC—Predicted pMIC.

**Table 2:** Intercorrelation data of descriptors used to develop 2D QSAR Model.

Property	AlogP	HBA Count	LUMO Eigenvalue VAMP	Molecular Polar Surface Area
**AlogP**	1.000	−0.367	−0.264	−0.490
**HBA Count**		1.000	−0.114	0.405
**LUMO Eigenvalue VAMP**			1.000	−0.350
**Molecular Polar Surface Area**				1.000

**Table 3:** Regression statistics table.

R	r^2^	r^2^ (Adjusted)	r^2^ (Prediction)	RMS Residual Error	q^2^ (Cross-Validation)	RMS Residual Error (Cross-Validation)
0.856	0.732	0.705	0.613	0.399	0.562	0.526

**Table 4:** Regression statistics table.

R	r^2^	r^2^ (Adjusted)	r^2^ (Prediction)	RMS Residual Error	q^2^ (Cross-Validation)	RMS Residual Error (Cross-Validation)
0.882	0.777	0.753	0.721	0.375	0.690	0.447

**Table 5:** Statistical results of the 3D QSAR model.

SD	r^2^	r^2^ _CV_	r^2^ Scramble	Stability	F	P	RMSE	Q^2^	Pearson-r
0.356	0.805	0.568	0.482	0.883	56.4	1.31×10^−14^	0.52	0.528	0.835

**Table 6:** Field Distribution (%).

**Steric**	**Electrostatic**	**Hydrophobic**	**H-Bond Acceptor**	**H-Bond Donor**
36.1	9.4	29.8	9.8	14.9

## Data Availability

Supporting information for this article is available upon request from corresponding authors.

## Supplementary Materials

The following supporting information can be downloaded at: https://www.mdpi.com/article/10.3390/ijms23084085/s1.
