# Supplementary material for: QSAR, Docking, and Molecular Dynamics Simulation Studies of Sigmacidins as Antimicrobials against Streptococci
Source: Int J Mol Sci. 2022 Apr 7;23(8):4085. doi: 10.3390/ijms23084085 (PMC9025105; doi:10.3390/ijms23084085)
Supplement: Supplementary file 1 [file ijms-23-04085-s001.zip › ijms-1653397-supplementary.pdf]

Supporting Information

QSAR, Docking, and Molecular Dynamics Simulation Studies of Sigmacidins as Antimicrobials against *Streptococci*

Jiqing Ye [a], Xiao Yang [b,*], Cong Ma [a,*]

[a] State Key Laboratory of Chemical Biology and Drug Discovery, and Department of Applied Biology and Chemical Technology, The Hong Kong Polytechnic University, Kowloon, Hong Kong SAR, China

[b] Department of Microbiology, The Chinese University of Hong Kong, Prince of Wales Hospital, Shatin, Hong Kong SAR, China

* Corresponding authors, E-mail: cong.ma@polyu.edu.hk (C.M.); xiaoyang@cuhk.edu.hk (X.Y.)

Table S1. Chemical structures of the antimicrobials

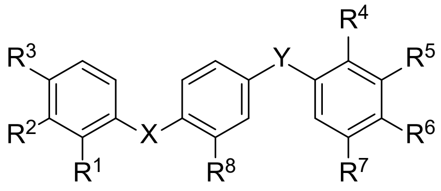

Formular structure of **1~56, except cpd. 5 and 22**

Index	R^1	R^2	R^3	R^4	R^5	R^6	R^7	R^8	X	Y
**1**	NH_2	H	H	COOH	H	H	H	NO_2	S	C(=O)
**2**	OCH_3	H	H	COOH	H	H	H	NO_2	S	C(=O)
**3**	H	Cl	Cl	COOH	H	H	H	NO_2	S	C(=O)
**4**	H	H	H	COOH	H	H	H	NO_2	NH	C(=O)
**5**	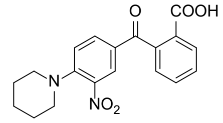									
**6**	NH_2	H	H	H	COOH	H	H	NO_2	S	C(O)NH
**7**	NH_2	H	H	H	COOH	H	H	NO_2	S	CH_2NH
**8**	H	NH_2	H	COOH	H	H	H	NO_2	S	C(=O)
**9**	H	H	NH_2	COOH	H	H	H	NO_2	S	C(=O)
**10**	NO_2	H	$CONEt_2$	COOH	H	H	H	NO_2	S	C(=O)
**11**	H	H	H	COOH	H	H	H	NO_2	CH_2NH	C(=O)
**12**	F	H	H	COOH	H	H	H	NO_2	CH_2NH	C(=O)
**13**	H	CH_3	H	COOH	H	H	H	NO_2	NH	C(=O)
**14**	H	Cl	Cl	H	COOH	H	H	NO_2	S	CH_2NH
**15**	H	Cl	Cl	H	COOH	H	H	NO_2	S	C(O)NH
**16**	H	t-Bu	H	COOH	H	H	H	NO_2	NH	C(=O)
**17**	F	H	H	COOH	H	H	H	NO_2	S	C(=O)
**18**	H	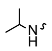	H	COOH	H	H	H	NO_2	S	C(=O)
**19**	NH_2	H	H	H	H	COOH	H	NO_2	S	C(O)NH
**20**	NH_2	H	H	COOH	H	H	H	NO_2	S	C(O)NH
**21**	CH_3	H	CH_3	COOH	H	H	H	NO_2	S	C(=O)
**22**	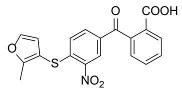									
**23**	Cl	H	Cl	COOH	H	H	H	NO_2	S	C(=O)
**24**	H	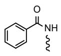	H	COOH	H	H	H	NO_2	S	C(=O)
**25**	NH_2	H	Cl	COOH	H	H	H	NO_2	S	C(=O)
**26**	H	Cl	Cl	COOH	H	H	H	NO_2	NH	C(=O)

**27**	NH_2	H	H	COOH	H	H	H	NO_2	S	CH_2NH
**28**	NH_2	H	H	H	H	COOH	H	NO_2	S	CH_2NH
**29**	H	AcNH	H	COOH	H	H	H	NO_2	S	C(=O)
**30**	H	Cl	Cl	Cl	H	H	COOH	NO_2	S	CH_2NH
**31**	H	Cl	Cl	OCH_3	H	H	COOH	NO_2	S	CH_2NH
**32**	H	Cl	Cl	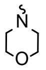	H	H	COOH	NO_2	S	CH_2NH
**33**	H	Cl	Cl	H	SO_2NH_2	H	H	NO_2	S	CH_2NH
**34**	H	Cl	Cl	CH_3	H	H	COOH	NO_2	S	CH_2NH
**35**	H	Cl	H	COOH	H	H	H	NO_2	NH	C(=O)
**36**	H	Cl	Cl	Br	H	H	COOH	NO_2	S	C(=O)
**37**	H	Cl	Cl	H	COOH	Cl	H	NO_2	S	C(=O)
**38**	H	Cl	Cl	COOH	H	H	H	NO_2	S	CH_2NH
**39**	H	Cl	Cl	COOH	H	H	H	H	S	C(=O)
**40**	H	Cl	Cl	COOH	H	H	Cl	NO_2	S	CH_2NH
**41**	H	Cl	Cl	COOH	H	H	H	NO_2	S	CH_2
**42**	H	Cl	Cl	COOH	H	H	H	NH_2	S	C(=O)
**43**	H	Cl	Cl	COOH	H	H	H	NMe_2	S	C(=O)
**44**	H	Cl	Cl	COOH	H	H	Cl	NO_2	S	C(=O)
**45**	H	Cl	Cl	COOH	H	Cl	H	NO_2	S	C(=O)
**46**	H	Cl	Cl	COOH	H	H	CF_3	NO_2	S	C(=O)
**47**	H	Cl	Cl	COOH	H	Cl	H	NO_2	S	CH_2
**48**	H	Cl	Cl	COOH	H	H	CH_3	NO_2	S	CH_2
**49**	H	Cl	Cl	COOH	H	H	OCH_3	NO_2	S	C(=O)
**50**	H	Cl	Cl	COOH	H	H	F	NO_2	S	C(=O)
**51**	H	Cl	Cl	COOH	H	CF_3	H	NO_2	S	C(=O)
**52**	H	Cl	Cl	COOH	H	H	CF_3	NO_2	S	CH_2
**53**	H	Cl	Cl	COOH	H	H	OCH_3	NO_2	S	CH_2
**54**	H	Cl	Cl	COOH	H	H	Cl	NO_2	S	CH_2
**55**	H	Cl	Cl	COOH	H	H	F	NO_2	S	C(=O)
**56**	H	Cl	Cl	COOH	H	CF_3	H	NO_2	S	C(=O)
